# Moral labels increase cooperation and costly punishment in a Prisoner’s Dilemma game with punishment option

**DOI:** 10.1038/s41598-021-89675-6

**Published:** 2021-05-13

**Authors:** Laura Mieth, Axel Buchner, Raoul Bell

**Affiliations:** Department of Experimental Psychology, Heinrich Heine University Düsseldorf, Universitätsstrasse 1, 40225 Düsseldorf, Germany

**Keywords:** Psychology, Human behaviour

## Abstract

To determine the role of moral norms in cooperation and punishment, we examined the effects of a moral-framing manipulation in a Prisoner’s Dilemma game with a costly punishment option. In each round of the game, participants decided whether to cooperate or to defect. The Prisoner’s Dilemma game was identical for all participants with the exception that the behavioral options were paired with moral labels (“I cooperate” and “I cheat”) in the moral-framing condition and with neutral labels (“A” and “B”) in the neutral-framing condition. After each round of the Prisoner’s Dilemma game, participants had the opportunity to invest some of their money to punish their partners. In two experiments, moral framing increased moral and hypocritical punishment: participants were more likely to punish partners for defection when moral labels were used than when neutral labels were used. When the participants’ cooperation was enforced by their partners’ moral punishment, moral framing did not only increase moral and hypocritical punishment but also cooperation. The results suggest that moral framing activates a cooperative norm that specifically increases moral and hypocritical punishment. Furthermore, the experience of moral punishment by the partners may increase the importance of social norms for cooperation, which may explain why moral framing effects on cooperation were found only when participants were subject to moral punishment.

## Introduction

Within Economics and Economic Psychology, social dilemma games such as the Ultimatum game^1^, the Public Goods game^2^ and the Prisoner’s Dilemma game^3^ are used to break down the complexities of human social interactions into specific payoff structures. Costs and benefits are often communicated to the participants in strictly numerical terms to examine how human behavior changes as a function of monetary incentives. However, human behavior does not fully conform to the predictions of a homo-oeconomicus model and is thus not only determined by monetary incentives but also by moral norms. Therefore, the behavior under investigation can be expected to be strongly influenced by perceptions of moral acceptability^4^. For framing effects [e.g.^5^], it has been demonstrated that defection in the Prisoner’s Dilemma game is seen as morally unacceptable or morally adequate depending on the name of the game (e.g., “Community Game” or “Wall Street Game”). Furthermore, verbal labels that emphasize the moral nature of social dilemmas have strong effects on the participants’ choices^6,7^, suggesting that human behavior is not only under the control of the payoff contingencies but also heavily influenced by the moral interpretation of the behavioral options. Following and extending this line of research, the present study was designed to investigate the effects of verbal labels on cooperation in a Prisoner’s Dilemma game in which a costly punishment option was included to test whether the enforcement of cooperation through punishment is affected by moral framing.

Cooperation is an interaction between two or more individuals in which one individual helps another^8^. Cooperation increases the inclusive fitness of the helper when cooperation is directed towards relatives^9^. However, cooperation can increase the individual’s chances of survival and reproduction even when it is directed towards non-relatives. While at first glance accepting costs to help non-relatives may seem incompatible with Darwin’s^10^ struggle for existence, helping non-relatives can be beneficial for one’s own survival in the long run when the support is reciprocated at a later time, provided that the helper’s cost is smaller than the other individual’s benefit^11^. Trivers’ original example is that of a drowning *Person X* who has a chance of 0.50 to survive without help. *Person Y* has the opportunity to rescue *X* but the chance of *Y* drowning is 0.20. If *Y* rescues *X* successfully, it is assumed that *X* reciprocates at a later point. If the situation is exactly the other way around, both *X* and *Y* reduce their individual chances of drowning from 0.50 to 0.10 (when assuming, for simplicity, that rescue attempts are always successful and other costs are negligible). The theory of reciprocal altruism is thus able to explain why it can be beneficial for non-relatives to cooperate with each other. However, an important condition has to be met: *X* has to reciprocate by supporting *Y* at some point in the future. This creates a free-rider problem because *X* may shy away from the costs of reciprocating. It is important to solve the free-rider problem to create a circle of mutual reciprocation. This may be accomplished by excluding cheaters from social exchange or by enforcing cooperation through punishment^12,13^.

The dynamics of cooperation can easily become quite complex when people cooperate in large groups^14^. Social and internalized norms can help to sustain cooperation in situations in which each individual has to invest resources to achieve a common goal^15^. Norms are prescriptions or proscriptions of actions among the members of a social system. Prescriptive and proscriptive norms are often enforced by punishing norm violations or rewarding norm-abiding behaviors^16^. Punishment can be seen as a solution to the free-rider problem because punishment imposes costs on free-riders that eliminate the incentives of free riding^17^. Consistently applied punishment may reduce the net costs of punishment because free-riding loses its appeal and, in consequence, may become less prevalent so that there is less need for punishment^18^. However, punishment is never without costs. For example, punishing others includes the risk of retaliation^19,20^. This may create a second-order free-riding problem: people may shy away from the costs of punishing. Moral punishment can thus be seen as a form of second-order cooperation^21^. It follows that moral punishment should be under the control of moral norms, just like first-order cooperation.

**Figure 1:**
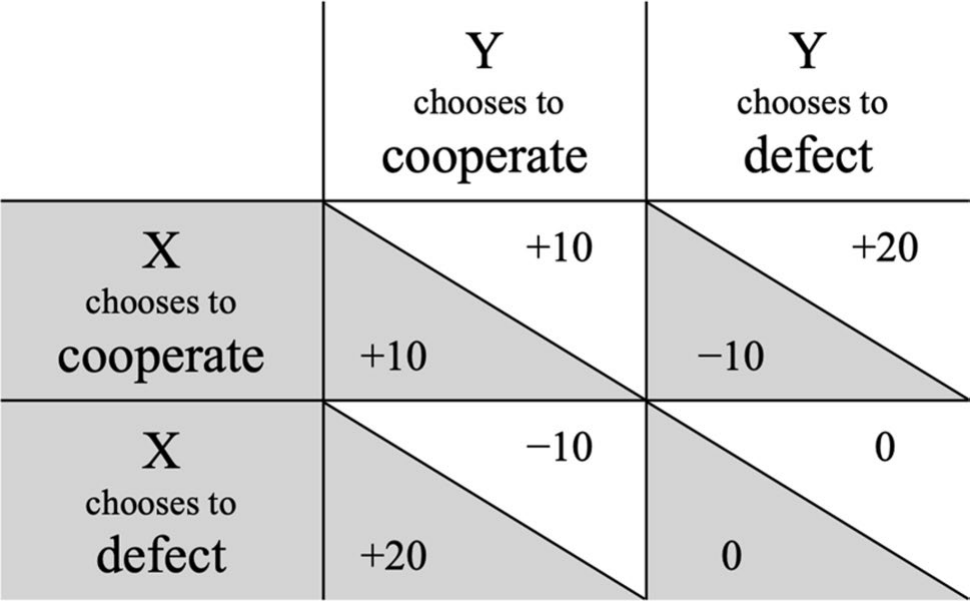
An example for the payoff matrix of a Prisoner’s Dilemma game (in cents). Values on gray backgrounds represent the payoffs of *Player X* while values on white backgrounds represent the payoffs of *Player Y*. The Prisoner’s Dilemma game is characterized by a payoff structure in which unilateral defection yields the highest payoff, followed by mutual cooperation which, in turn, yields a higher payoff than mutual defection. Unilateral cooperation yields the lowest payoff.

Social dilemma games are defined by their specific payoff structures^22^. The Prisoner’s Dilemma game^3^ is a model of a simple interaction between two individuals who have to choose whether to cooperate or to defect (Fig. 1). Within this game, unilateral defection yields the highest payoff, followed by mutual cooperation which, in turn, yields a higher payoff than mutual defection. Unilateral cooperation yields the lowest payoff. This payoff structure implies that, at a collective level, cooperation is desirable because the net payoff for both players combined is higher when both players cooperate than when they defect. However, these collective interests clash with the individual interests of the players. For each individual player, defection is always associated with a higher payoff than cooperation, regardless of what the other player does. In a world of selfish individuals, cooperation is doomed to fail^23^. Given that the Prisoner’s Dilemma game is a model of real-world situations with similar conflicts between collective and individual interests^22^, it is highly desirable to know how cooperation can be achieved even under these adverse circumstances.

Given that game-theoretical paradigms have been developed to examine the influence of incentives on behaviors, verbal labels that facilitate a social interpretation of the behavioral options are often avoided because they are seen as extraneous influences that may introduce unwanted biases. Therefore, the behavioral options that stand for cooperation and defection are often labeled with meaningless digits (such as “1” and “2”) or letters (such as “A” and “B”) to avoid distracting participants from the incentive structure of the game [e.g.^3,24,25^]. While it is certainly interesting to examine how people respond to raw monetary incentives, people’s behavior is not always directed at maximizing monetary rewards. Specifically, there are strong norms for cooperation and against defection that may play an important role in shaping people’s decisions towards cooperation. In many situations, cooperation is seen as desirable while in others competition is expected. Moral context framing through verbal labels is one way to examine the influence of moral norms^5,26^. In the absence of an explicit moral framing, participants may create an interpretative frame on their own that may deviate to some unknown degree from that of the researcher. Zhong et al.^7^ recently made aware of this issue: “In essence, the games that we felt that we were studying may have been systematically different from the games that our participants thought that they were playing. Thus, PD [Prisoner’s Dilemma game] researchers and participants may not have been thinking or speaking the same language as they have interpreted the games, the choices, and the outcomes” (p. 433). Explicating a certain social situation and using clear verbal labels for the behavioral options may facilitate the construction of a coherent social context, provide transparency, and may help to align the interpretations of participants and researchers. At the same time, it is clear that such a framing may change the very nature of the game and shape the participants’ behaviors accordingly.

Decisions in Prisoner’s Dilemma games are determined by multiple factors [for a meta-analysis see^27^]. Among those, verbal labels can be used to manipulate the interpretation of various aspects of social dilemma games: it is possible to assign verbal labels to the games themselves [e.g.^28^], to the behavioral options [e.g.^5,6,29^] and to the outcomes [e.g.^6,7^]. In most studies, moral labels increased cooperation, but there are also some studies showing no effects or even negative effects. In an early study on this topic^6^, participants were less willing to cooperate when the behavioral options in a Prisoner’s Dilemma game were labeled “cooperation” and “competition” in comparison to when they were labeled with letters. The authors interpreted their findings as suggesting that the verbal labels may have improved participants’ understanding of the payoff structure of the game, leading to more strategic and rational choices. However, studies showing negative effects of moral labels on cooperation are the exception rather than the rule. Rege and Telle^30^ showed that instructions emphasizing a social interpretation of a Public Goods game increased cooperation, but the effect did not reach the conventional level of statistical significance, possibly due to the small sample size of the study. In a study by Zhong et al.^7^, a Prisoner’s Dilemma game was labeled “cutthroat game” or “trust game”. These moral-framing conditions were compared to a control condition with neutral labels. Even though the majority of the participants stated that they had not attended to the labels, the moral labels significantly increased their willingness to cooperate in comparison to the neutral control condition. In a second experiment, moral framing led to increased expectations of others’ cooperation. Ellingsen et al.^31^ as well as Dreber et al.^32^ found that framing effects were more prevalent in Prisoner’s Dilemma games in which there was an uncertainty about the partner’s behavior than in games with no such uncertainty. These findings were interpreted as suggesting that moral framings affect cooperation by changing the participants’ expectations about their partners’ behaviors.

In comparison to the bulk of evidence showing effects of moral framing on cooperation^5–7,26–28,30–32^, the effect of moral framing on punishment is less well understood and the few studies that are available provide inconsistent findings. Cubitt et al.^33^ as well as McCusker and Carnevale^34^ examined the influence of a “give” or “take” framing on the participants’ willingness to punish deviating behavior in a Public Goods game. Both studies consistently reported no effects of framing on punishment. However, in stark contrast to these findings, there is evidence suggesting that framing affects the rejection rate in the Ultimatum game. Specifically, Marchetti et al.^35^ found that describing the proposer in an Ultimatum game as a “selfish” person increased the rejection rate of the proposer’s offer. Given that the rejection of unfair offers in the Ultimatum game can be seen as a form of costly punishment^36^, this finding suggests that costly punishment is affected by the social construction of the situation in which it is applied. It thus seems possible that moral framing affects punishment in a similar way as it affects cooperation: costly punishment should increase when verbal labels suggest a moral interpretation of the social dilemma. However, the few studies that are currently available are insufficient to draw clear conclusions, and a more direct approach towards studying the effects of moral framing on costly punishment seems desirable. The present study primarily serves to expand the existing literature on framing effects by focusing on the effects of moral framing on costly punishment. Framing was manipulated by using moral labels (“I cooperate” and “I cheat”) or neutral labels (“A” and “B”) for cooperation and defection in the Prisoner’s Dilemma game with a punishment option. “I cooperate” and “I cheat” were chosen as moral labels because they have strong moral connotations and thus should activate norms of cooperation. In order to measure the effects of the labels on cooperation and punishment, we used a simultaneous Prisoner’s Dilemma game with a punishment option that has already been successfully used by Mieth et al.^37,38^ to examine the effects of facial expression and facial gender on cooperation and punishment. One advantage of this paradigm is that it allows us to distinguish moral, hypocritical and antisocial punishment from a general punishment bias (see “Measuring cooperation and punishment” section).

*Moral punishment* is directed at a unilaterally defecting partner. This type of punishment is most strongly connected to social norms. Its assumed purpose is to reinforce cooperative norms^17,39^. Therefore, the directional hypothesis can be derived that moral punishment should be strongly influenced by framing: moral punishment should be higher in the moral-framing condition than in the neutral-framing condition. Moral punishment is the most common type of punishment, but there are other types of punishment as well. For instance, people are known to punish the defection of a partner even when they have defected themselves. *Hypocritical punishment*^40^ following mutual defection has been attributed to spitefulness^41^. If people are simply motivated by a malicious desire to harm their partners or to maximize the difference between their own and their partner’s payoff, the moral-framing manipulation should fail to have an effect on hypocritical punishment. However, it is also possible to postulate that hypocritical punishment is caused by a truly hypocritical moral outrage about the partner’s defection. If punishment is, in fact, hypocritical in the strict sense of the word, then it should be amplified when the labels activate moral norms. *Antisocial punishment*^42,43^ occurs when a defector punishes a cooperator. A priori, it is unclear whether antisocial punishment depends on moral norms. If antisocial punishment is an expression of the opposition against norms of cooperation, then antisocial punishment may well be affected by the moral labels. However, if antisocial punishment is primarily determined by asocial motivations such as the motivation to maximize the difference to the partner’s payoff or to hurt the partner, then it should remain unaffected by the framing. Finally, given that there is no reasonable justification for punishing mutual cooperation, it has been proposed that any residual punishment in this condition is caused by an unspecific *bias to punish*, and thus provides a baseline against which the other types of punishment can be compared^38^. A priori, we expected moral labels to affect those types of punishment that are specifically directed at the enforcement of social norms. Therefore, the moral-framing manipulation was not expected to affect the general punishment bias.

To summarize, conflicting hypotheses can be derived about the effect of moral labels on cooperation. On the one hand, it can be hypothesized that moral framing should activate cooperative norms. Based on this, cooperation should increase in the moral-framing condition, provided that cooperation is under the control of moral norms^7^. However, framing effects on cooperation have not always been found^24^. It has even been argued that verbal labels may help participants to understand the incentive structure of social-dilemma games, which may lead to decisions that are more rational and, in consequence, less cooperative^6^. The present study can provide further insight into the extent to which moral labels affect cooperation (a) when participants have the opportunity to punish their partners but do not have to fear punishment themselves (Experiment 1) and (b) when participants are morally punished by their partners (Experiment 2). However, the main purpose of the present study is to examine the effects of moral framing on punishment. A directional hypothesis can be formulated with respect to moral punishment: if *moral punishment* is not a misnomer, then verbal labels that lead to a moral interpretation of the behavioral choices in the social dilemma game should increase moral punishment in comparison to a condition with neutral labels. *Hypocritical* and *antisocial punishment* should be affected by the moral labels only to the degree to which these types of punishment are based on moral norms. Finally, specific types of punishment have to be distinguished from an unspecific bias to punish^38^, the latter of which should not be affected by framing.

## Experiment 1

### Method

#### Participants

Ninety-eight participants (73 female, 25 male) with a mean age of 22 (*SD*_Age_ = 3) took part in the study. A sensitivity analysis in G*Power^44^ showed that it was possible to detect a small effect of size *w* = 0.08^45^ when comparing the punishment parameters between the condition with moral labels and the condition with neutral labels, given α = β = 0.05, 20 interactions per participant, and a sample size of *N* = 98 participants. After the experiment was completed, all participants received the money in their account balance (see below) and either a participation honorarium of 4 € or course credit.

#### Ethics statement

All participants gave written informed consent in accordance with the Declaration of Helsinki. Before the experiment, participants were not explicitly made aware of the fact that they played with computer-controlled partners, but they were fully debriefed at the end of the study. The study was approved by the ethics committee of the Faculty of Mathematics and Natural Sciences of Heinrich Heine University Düsseldorf.

#### Materials, procedure, and design

A 2 × 2 design was used with framing (moral, neutral) as between-subjects factor and partner behavior (defection, cooperation) as within-subjects factor.

For each participant, 20 pictures of faces of their own gender were randomly selected without replacement from a pool of 64 female and 62 male young white adults that had been photographed in frontal view with a neutral facial expression for the Center for Vital Longevity Face Database^46^. The pictures were presented at a resolution of 640 × 480 pixels.

**Figure 2:**
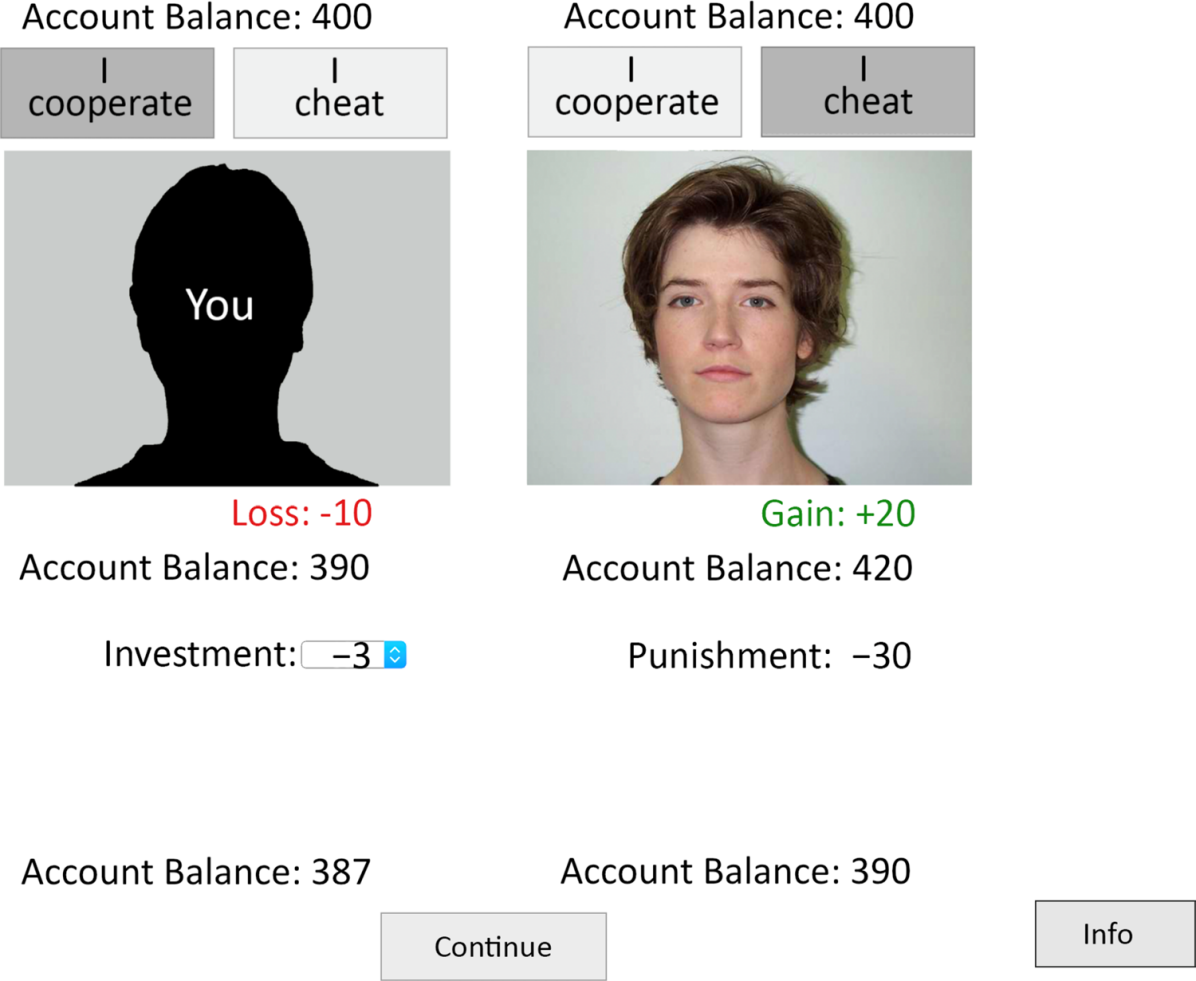
A screenshot of the simultaneous Prisoner’s Dilemma game with the costly punishment option used in Experiment 1. Depending on the framing condition, the grey buttons above the pictures displayed either the moral labels “I cooperate” and “I cheat” or the letters “A” and “B”. In the example trial displayed here, the participant cooperated while the partner cheated. The participant then chose to punish the partner by investing 3 cents to reduce the partner’s account balance by 30 cents. The facial photographs on the right side (one unique photograph for each partner) were taken from the Center for Vital Longevity Face Database^46^.

As in previous studies^37,38^, participants played a Prisoner’s Dilemma game with a costly punishment option (see Fig. 2). Participants were alternately assigned to one of two conditions. In the condition with moral labels, the Prisoner’s Dilemma game required participants to choose between the options “I cooperate” and “I cheat”. We deliberately chose morally loaded labels in the moral-framing condition to increase the difference to the neutral-framing condition. We also took care to select words that are commonly used in everyday language so that the labels are easy to understand for the participants without training in game theory. In the condition with neutral labels, the participants had to choose between the options “A” and “B”. Apart from these labels, the games were exactly identical in both groups.

At the start of the experiment, all participants were informed that they had 400 cents at their disposal that they could use to play the Prisoner’s Dilemma game and to apply costly punishment. They were informed that the monetary incentives were real and that they would receive the money in their account balance in addition to either a participation honorarium of 4 € or course credit at the end of the experiment. On average, the final account balance was 514 cents (*SD* = 66). In the instructions, the Prisoner’s Dilemma game was explained to the participants. The payoff matrix of this game was the one shown in Fig. 1. Depending on the condition, the payoff matrix was presented with the moral labels or with the neutral labels. During the Prisoner’s Dilemma game, participants could revisit the payoff matrix by clicking the “Info” button in the lower right of the screen. After having been informed about the payoffs in the instructions, 48% of the participants did not feel the need to do so, another 46% of the participants clicked the “Info” button 1 to 3 times, and only 6% of the participants clicked it more than 3 times. Participants in both conditions clicked the “Info” button equally often, χ^2^(8) = 7.11, *p* = 0.525.

The participant was represented by a silhouette on the left side of the screen. In each trial, participants played with a different partner so that they played with each partner only once. The face of their current partner was shown on the right side of the screen. The partner’s account balance was at most 10 cents above or below that of the participant. In the moral-framing condition, the participant chose between the options “I cooperate” or “I cheat”. In the neutral-framing condition, the participant chose between the options “A” or “B”. The game was a simultaneous Prisoner’s Dilemma game which implies that the participant’s choice and the partner’s choice were displayed at the same time. The choices remained highlighted for the entire trial. After 5 s, the participant’s gain or loss and the partner’s gain or loss were displayed on the screen. As shown in Fig. 1, unilateral defection yielded the highest payoff (20 cents), mutual cooperation yielded a higher payoff (10 cents) than mutual defection (0 cents) and unilateral cooperation yielded a loss (− 10 cents). After 5 s, the updated account balances were displayed.

One second later, the punishment option was available to the participant. The participants either selected the “0 cents” option for no punishment or they could spend 1 to 9 cents for the costly punishment option to reduce their partner’s payoff by 10 to 90 cents. For each cent they invested, 10 cents were subtracted from their partners’ account balances. The choice was confirmed by clicking on the “Punishment” button. In Experiment 1, punishment was unilateral. The participants could punish the partners but the partners could not punish the participants. The updated account balances showing the effects of the participants’ investments and of the punishments of the partners were shown at the bottom of the screen. The participants could then initiate the next round by clicking on the “Continue” button.

As is common in Economic Psychology [e.g.^7,47^], participants played with partners whose choices were randomly determined by a computer program with the restriction that a randomly selected half of the partners cooperated while the other half cheated. This served to increase experimental control over the partners’ behaviors. Note that the randomization procedure implies that a face that was associated with cheating for one participant could be associated with cooperation for another participant.

At the end of the experiment, all participants received the money they had in their account balance after the last round of the game. In addition, they were thanked, debriefed, and compensated for their participation.

#### Measuring cooperation and punishment

To assess costly punishment, it is important to distinguish between different types of punishment that may occur as a function of the partner’s and the participant’s choices in the Prisoner’s Dilemma game. Furthermore, it seems desirable to distinguish between the effect of the manipulation on a specific form of punishment (e.g., moral punishment) and a general bias to punish the partners regardless of the outcome of the Prisoner’s Dilemma game. To achieve this, we used a formal measurement model. Multinomial processing tree (MPT) models are commonly used in Cognitive Psychology to estimate probabilities of latent cognitive states from observable behaviors^48,49^.

**Figure 3:**
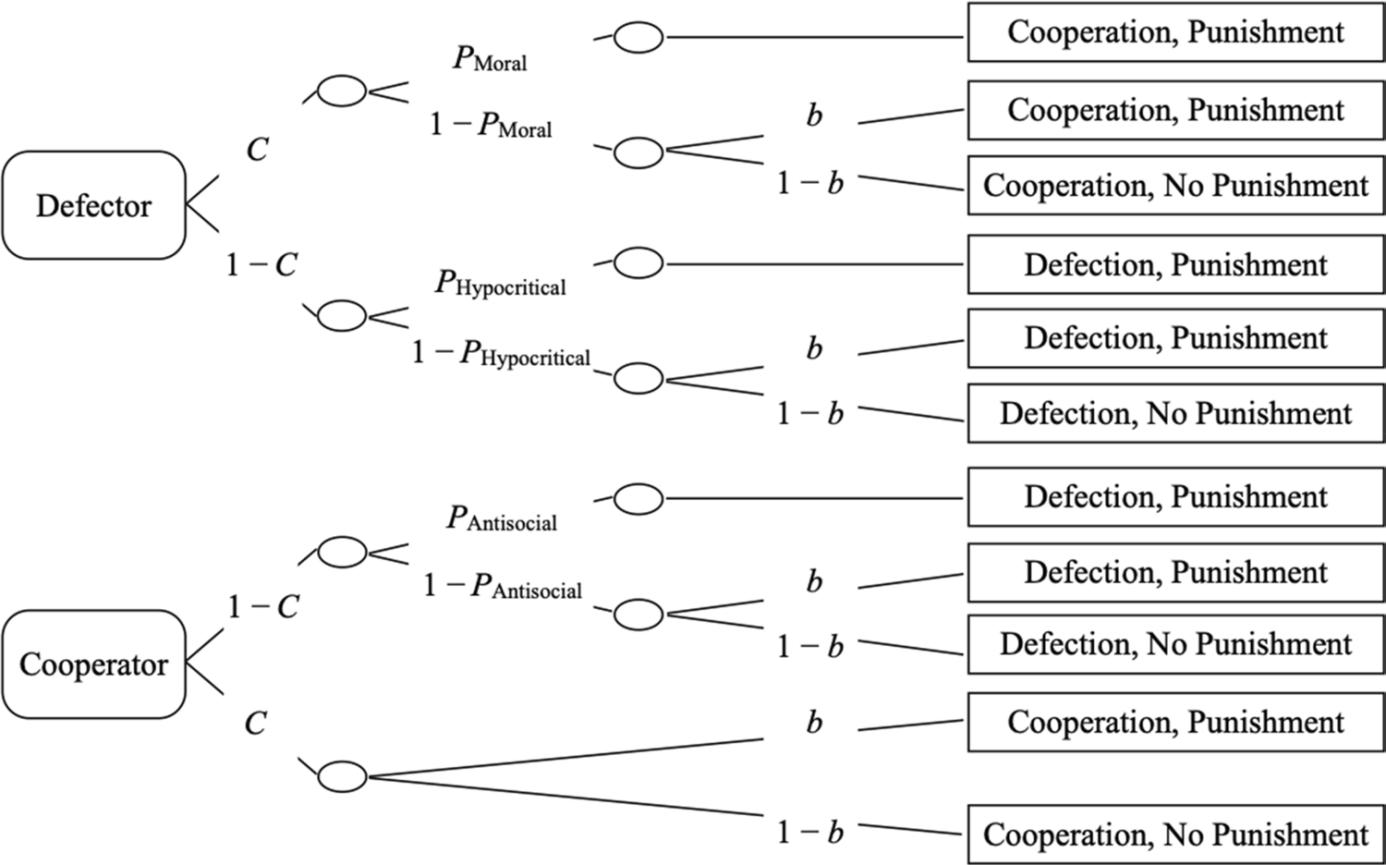
The multinomial model of cooperation and punishment. Rounded rectangles on the left side represent the partners’ behaviors in the Prisoner’s Dilemma game. Rectangles on the right represent the participants’ behaviors. The model was used to estimate the probability of the participants’ cooperation (*C*), conditional probabilities for different types of punishment (*P*_Moral_, *P*_Hypocritical_, and *P*_Antisocial_), and the participants’ bias to punish (*b*).

The cooperation-and-punishment model displayed in Fig. 3 is an updated version of a model that was successfully applied to measure cooperation and punishment in previous studies^37,38^. The rounded rectangles on the left represent the choices of the two partners in the Prisoner’s Dilemma game. The rectangles on the right represent the participant’s choices. The letters along the branches represent probabilities that vary between 0 and 1. In a first step, the participant decides whether to cooperate, with probability *C*, or to defect, with the complementary probability 1 − *C*. This choice is assumed to be independent of that of the partner because the choices of both partners are revealed simultaneously so that the participants do not know, at the time they decide to cooperate or to defect, whether their partners will decide to cooperate or to defect. When a participant decides to cooperate and the partner decides to defect, the participant may choose to morally punish the partner with probability *P*_Moral_. Within the model, moral punishment is defined as punishment that specifically serves to decrease the partner’s payoff after the partner’s unilateral defection. However, if the participant does not apply moral punishment with probability 1 − *P*_Moral_, the participant may still apply punishment because of a general punishment bias *b* that is assumed to be independent of the specific outcome of the Prisoner’s Dilemma game. Alternatively, with probability 1 − *b*, no punishment is applied. Following mutual defection, the participant may punish the partner hypocritically with probability *P*_Hypocritical_. With probability 1 − *P*_Hypocritical_, no punishment is applied unless the participant has a general bias to punish the partner. When a participant has defected against a cooperating partner, the participant shows antisocial punishment with probability *P*_Antisocial_. With probability 1 − *P*_Antisocial_, no punishment is applied unless the participant has a general bias to punish the partner. Unilateral cooperation is special because there is no reason to assume that a participant specifically punishes mutual cooperation^38^. Therefore, the model assumes that any residual punishment in this condition is caused by the punishment bias *b*. The underlying logic is thus parallel to other areas of research where the participants’ responses are corrected for response bias^49,50^.

*MultiTree*^51^ was used to verify the identifiability of the model, to estimate the parameters from the response frequencies and to perform goodness-of-fit tests. We needed two sets of the trees depicted in Fig. 3 to analyze the present data sets, one for the condition with moral labels and one for the condition with neutral labels. Hypotheses tests were performed by testing whether the parameters could be constrained to be equal across both framing conditions (moral, neutral). This was done by assessing the difference in goodness of fit between the restricted model and the unrestricted model.

### Results

**Figure 4:**
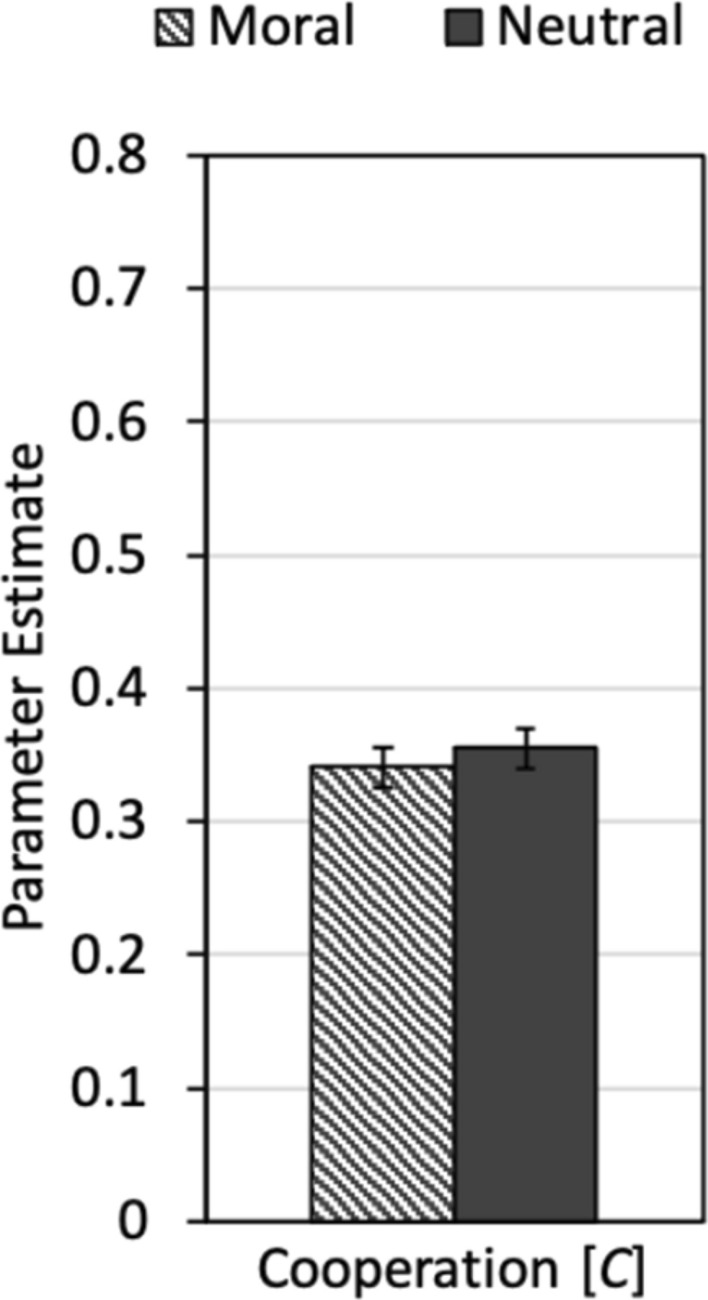
Parameter estimates of parameter *C* reflecting the probability that a participant decided to cooperate as a function of framing (moral, neutral). The error bars represent standard errors.

To disentangle cooperation and the different types of punishment, we used the multinomial model shown in Fig. 3. The cooperation-and-punishment model described above (without any additional constraints) fit the data well, *G*^2^(2) = 0.32, *p* = 0.850. Parameter estimates for the cooperation parameter *C* in each of the two labeling conditions are shown in Fig. 4. Cooperation did not differ between the conditions with moral labels and the condition with neutral labels, Δ*G*^2^(1) = 0.44, *p *= 0.507, *w *= 0.01.

**Figure 5:**
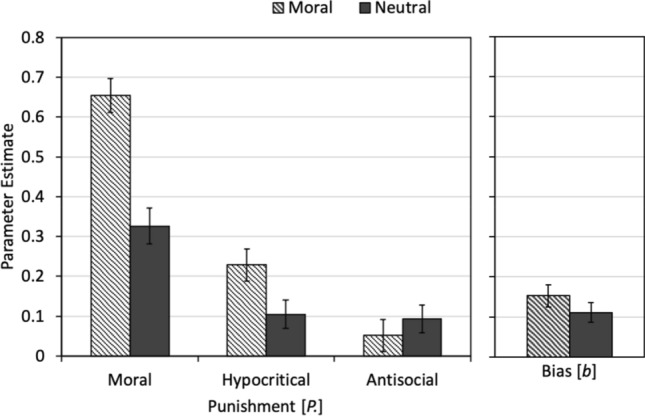
Estimates of the parameters reflecting moral punishment (*P*_Moral_), hypocritical punishment (*P*_Hypocritical_) and antisocial punishment (*P*_Antisocial_) are displayed as a function of framing (moral, neutral) in the left panel. The estimate of the punishment bias (*b*) parameter is shown in the right panel. The error bars represent standard errors.

Estimates of the three punishment parameters *P*_Moral_, *P*_Hypocritical_, and *P*_Antisocial_, and the bias parameter *b* are shown in Fig. 5. Moral punishment was more prevalent in the moral-framing condition than in the neutral-framing condition, Δ*G*^2^(1) = 25.09, *p *< 0.001, *w *= 0.11. Moral framing also increased the probability of hypocritical punishment relative to the neutral framing, Δ*G*^2^(1) = 5.16, *p *= 0.023, *w *= 0.05. Antisocial punishment, by contrast, did not differ between the condition with moral labels and the condition with neutral labels, Δ*G*^2^(1) = 0.61, *p *= 0.433, *w *= 0.02. The punishment bias also did not differ between the condition with moral labels and the condition with neutral labels, Δ*G*^2^(1) = 1.31, *p *= 0.253, *w *= 0.03. The results thus suggest that moral framing does not have a general effect on punishment but specifically increases moral punishment and hypocritical punishment relative to a neutral-framing condition.

### Discussion

The main aim of the present study was to understand how moral framing affects costly punishment. The directional hypotheses that moral framing should increase moral punishment relative to a neutral-framing condition was confirmed. Moral punishment was more prevalent when the behavioral options in the Prisoner’s Dilemma game were associated with moral labels (“I cooperate”, “I cheat”) than when they were associated with neutral labels (“A”, “B”), suggesting that the moral-framing manipulation was successful in shaping the moral interpretation of the Prisoner’s Dilemma game. An interesting observation is that the effect of framing was not restricted to moral punishment. Hypocritical punishment was higher in the moral-framing condition than in the neutral-framing condition too. This finding suggests that hypocritical punishment is not only motivated by the participant’s spiteful desire to hurt the partner or the desire to maximize the difference between their own payoff and that of their partner. Instead, hypocritical punishment may be at least partly motivated by a truly hypocritical outrage about the partner’s defection. Antisocial punishment, by contrast, remained unaffected by the presence or absence of the moral labels. This finding suggests that the effect of moral labels is specific for certain types of punishment while it is absent in others. This conclusion is further supported by the finding that the moral labels did not induce an unspecific punishment bias.

The participant’s willingness to cooperate remained unaffected by the verbal labels. This finding adds to previous research showing mixed effects of moral labels on cooperation. Some studies showed an increase in cooperation^7^, while others showed no effect^30^, or even a decrease of cooperation^6^ when moral-framing conditions were compared to neutral-framing conditions. These inconsistent findings suggest that the effects of moral framing on cooperation may depend on the specific nature of the social dilemma game, but it is difficult to pinpoint the specific factors responsible for the inconsistent results due to the large number of characteristics that differed among the studies. One of these characteristics is the punishment option that was added to the Prisoner’s Dilemma game in the present study. The influence of the punishment option is further examined in Experiment 2 in which moral punishment was symmetric (that is, available to both the participant and the partner).

## Experiment 2

In Experiment 2, we followed up on a specific aspect of the procedure of Experiment 1. In Experiment 1, punishment was asymmetric: The participants had the opportunity to punish their partners and enforce social norms of cooperation through moral punishment, but the punishment option was not available to the partners. It thus seemed interesting to test how moral labels influence the participants’ behaviors when participants experience moral punishment by their partners. Specifically, it has been proposed that cooperation may be more strongly determined by moral norms when punishment has to be feared^32^, which may increase the effects of moral framing on cooperation.

Another purpose of Experiment 2 was to test whether the effects of the moral labels on punishment would replicate. Specifically, moral punishment and hypocritical punishment should again be more likely when moral labels are used than when neutral letters are used to characterize the behavioral options in the Prisoner’s Dilemma game while antisocial punishment and the punishment bias should remain unaffected.

### Method

#### Participants

The sample in Experiment 2 consisted of 93 participants (57 female, 36 male) with a mean age of 24 years (*SD*_Age _= 4). A sensitivity analysis in G*Power^44^ showed that it was possible to detect a small effect of size *w *= 0.08^45^ when comparing the punishment parameters between the condition with moral labels and the condition with neutral labels, given α = β = 0.05, 20 interactions per participant, and a sample size of *N *= 93 participants. After the experiment was completed, all participants received the money in their account balance and either a participation honorarium of 4 € or course credit.

#### Materials, procedure, and design

Materials, Procedure, and Design of Experiment 2 were identical to those of Experiment 1 with the following exceptions. On average the final account balance was 236 cents (*SD *= 117). The partners had a punishment option which was visible in the interface and they were programmed to use this punishment option to morally punish the participants’ unilateral defection. When a participant cheated on a cooperating partner, the partner spent between 1 and 9 cents to subtract between 10 and 90 cents from the participant’s account. The amount of the punishment was randomly determined.

After having been informed about the payoff matrix in the instructions, 45% of the participants did not feel the need to use the “Info” button during the experiment at all, another 45% of the participants clicked it 1–3 times and only 10% of the participants clicked it more than three times. The “Info” button was used equally often in the condition with moral labels and the condition with neutral labels, χ^2^(9) = 9.47, *p *= 0.395.

### Results

**Figure 6:**
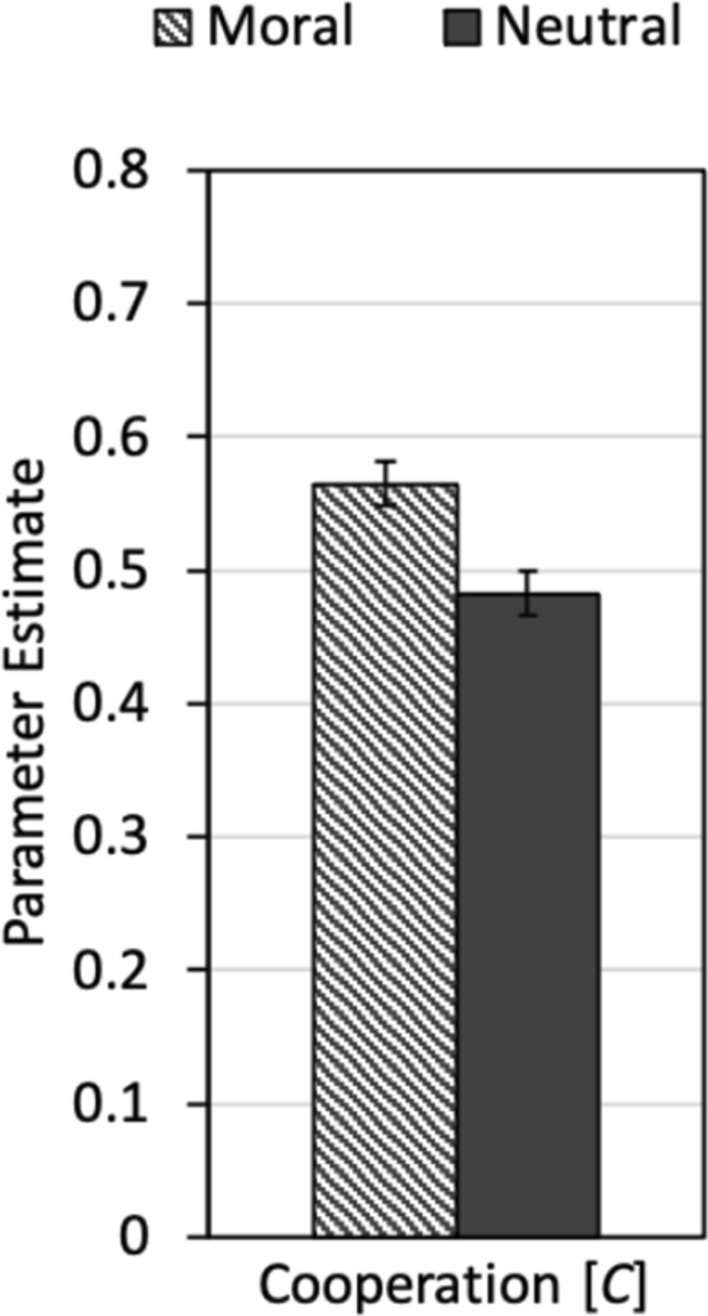
Parameter estimates for parameter *C* reflecting the probability that a participant decided to cooperate as a function of framing (moral, neutral). The error bars represent the standard errors.

To disentangle cooperation and the different types of punishment, we used the multinomial cooperation-and-punishment model shown in Fig. 3. The base model (without additional restrictions) fit the data well, *G*^2^(2) = 1.20, *p *= 0.548. Parameter estimates for the cooperation parameter *C* are shown in Fig. 6. In comparison to Experiment 1, the absolute level of cooperation is higher, suggesting that the fear of moral punishment increased the participants’ willingness to cooperate. More importantly, moral framing significantly increased the participants’ cooperation relative to the neutral-framing condition, Δ*G*^2^(1) = 12.64, *p *< 0.001, *w *= 0.08.

**Figure 7:**
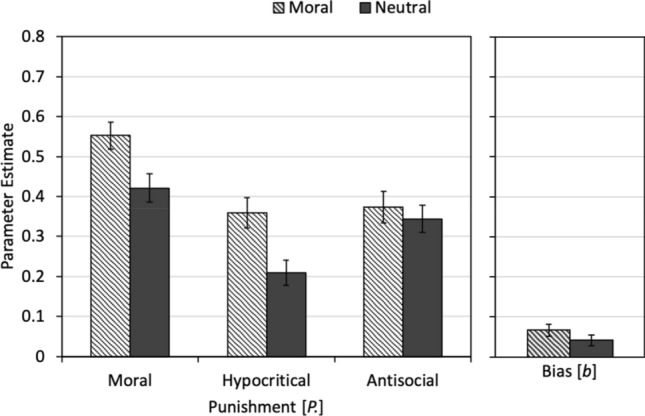
In the left panel, estimates of the punishment parameters reflecting moral punishment (*P*_Moral_), hypocritical punishment (*P*_Hypocritical_) and antisocial punishment (*P*_Antisocial_) are displayed as a function of framing (moral, neutral). In the right panel, the estimate of the punishment bias parameter (*b*) is displayed. The error bars represent the standard errors.

Estimates of the three punishment parameters *P*_Moral_, *P*_Hypocritical_, and *P*_Antisocial_, and the punishment bias parameter *b* are shown in Fig. 7. At an absolute level, the prevalence of the different punishment parameters are similar to those observed in Experiment 1, although antisocial punishment was somewhat higher than in Experiment 1 and punishment bias was somewhat lower [for a discussion of possible reasons for these changes, see^37,38^]. As in Experiment 1, moral punishment was more prevalent in the moral-framing condition than in the neutral-framing condition, Δ*G*^2^(1) = 7.19, *p *= 0.007, *w *= 0.06. Moral framing also increased the probability of hypocritical punishment relative to the neutral framing, Δ*G*^2^(1) = 9.15, *p *= 0.002, *w *= 0.07. By contrast, antisocial punishment did not differ between the conditions, Δ*G*^2^(1) = 0.33, *p *= 0.568, *w *= 0.01. The punishment bias also did not differ between the conditions, Δ*G*^2^(1) = 1.44, *p *= 0.231, *w *= 0.03.

### Discussion

The most important findings of Experiment 2 are that the effects of moral framing on moral punishment and hypocritical punishment observed in Experiment 1 were replicated. In line with our directional hypothesis, moral framing increased moral punishment relative to a neutral-framing condition. Hypocritical punishment increased in the moral-framing condition too, suggesting that hypocritical punishment is at least partly motivated by a truly hypocritical outrage about the partner’s violation of cooperation norms. As in Experiment 1, moral framing affected neither antisocial punishment nor the punishment bias. Moral framing thus did not lead to a general increase in the desire to punish. Instead, the moral framing effects were specific to moral and hypocritical punishment.

Interestingly, and in contrast to the results of Experiment 1, moral framing affected not only moral and hypocritical punishment but also the participants’ willingness to cooperate in the Prisoner’s Dilemma game. The main difference between Experiment 2 and Experiment 1 is that participants experienced moral punishment in Experiment 2 while they did not have to fear the partners’ punishment in Experiment 1. This fits with the conclusions of Dreber et al.^32^ who have proposed that cooperative behavior may be more under the control of cooperative norms when punishment has to be feared. The present results support this proposition. In the present study, the moral labels seem to have induced a moral interpretation of the Prisoner’s Dilemma game. However, this moral interpretation affected the willingness to cooperate in the Prisoner’s Dilemma game only when cooperation was enforced through punishment.

## General discussion

In human social life, a large variety of behaviors are regulated by social norms that set standards on how individuals should behave^52^. One of these norms is the norm of cooperation. In many situations, people are expected to set aside their egoistic interests to achieve the collective best outcome. Within economic research, cooperation is often studied in social dilemma games. In these games, the complexities of human social interactions are reduced to their incentive structures. However, human behavior is not *only* determined by monetary incentives. There are many other important determinants of behavior among which social norms are especially powerful. The participants’ decisions in social dilemma situations are thus affected by their interpretation of whether a certain behavior is socially appropriate or inappropriate. Moral labels can help to reduce the ambiguity of the social dilemma game by creating associations to real-life cooperation norms^30^. Thereby, the moral framing may support a moral interpretation of the social dilemma situation, resulting in the moral rejection of egoistic behaviors^7^. Often, social norms are enforced by punishment. It has been argued “that the maintenance of social norms typically requires a punishment threat, as there are almost always some individuals whose self-interest tempts them to violate the norm” [^52^, p. 185]. Given the strong link between social norms and moral punishment, we have postulated that verbal labels that facilitate a moral interpretation of the behaviors in the Prisoner’s Dilemma game should increase moral punishment. The present results confirm this hypothesis. In both experiments, participants were more likely to punish unilateral defection when moral labels were used to characterize the behavioral options in the Prisoner’s Dilemma game than when neutral labels were used. The moral labels may effectively communicate the norm that cooperation is morally desirable and defection is not acceptable, and moral punishment is used to enforce this norm. In the neutral control condition, the unilateral defection of the partner is characterized by the same payoff imbalance, but the labels of the behavioral options did not emphasize the moral dimension of the unilateral defection. The strong influence of framing on moral punishment suggests that a primary function of moral punishment is to enforce social norms of cooperation rather than to eliminate the payoff imbalance that results from the partner’s unilateral defection. Moral punishment is thus not only determined by the payoff structure, but also, to a substantial degree, by the moral interpretation of the partner’s behavior in the Prisoner’s Dilemma game.

While a directional hypothesis was formulated for moral punishment, it was a priori less clear whether hypocritical punishment would be affected by the moral labels. Hypocritical punishment is often attributed to a spiteful desire to harm the partner or to maximize the difference between one’s own payoff and that of the partner [e.g.^41^]. However, it is also possible that this type of punishment is hypocritical in the true sense of the word in that it may reflect the moral rejection of a perceived norm violation. The present results support the latter hypothesis as only this hypothesis implies that hypocritical punishment should be influenced by the moral labels. In both experiments, hypocritical punishment was more prevalent when defection in the Prisoner’s Dilemma game was labeled “cheating” than when it was labeled with a neutral letter. This finding suggests that this type of punishment is used to hypocritically enforce social norms.

At this point, it seems important to note that the presence or absence of moral labels did not affect all types of punishment equally. Most importantly, antisocial punishment did not differ between the moral-framing condition and the neutral-framing condition. This finding has been obtained consistently in both experiments. An obvious conclusion is that antisocial punishment is not directly under the control of the moral norms. Why should this be the case? It seems most plausible that antisocial punishment may best be understood as an aggressive act driven by the displeasure evoked by the mismatch between one’s own behavior and that of the partner. Note that moral framing also did not induce a general bias to punish the partner. This is important in that it shows that moral framing seems to specifically affect those types of punishment that serve to enforce moral norms of cooperation.

While the effects of the moral labels on the different types of punishment were quite consistent, the effect of the moral labels on cooperation differed between Experiments 1 and 2. This finding is interesting in that it suggests that the effects of moral framing on cooperation can depend on the enforcement of cooperation norms through punishment. Cooperation remained unaffected by framing in Experiment 1 but was higher in the moral-framing condition than in the neutral-framing condition in Experiment 2. The main difference between the two experiments was that participants’ unilateral defection was morally punished by their partners in Experiment 2 but not in Experiment 1. The pattern of findings suggests that the activation of a social norm is particularly effective in increasing cooperation when cooperation is enforced through moral punishment. This conclusion is compatible with previous findings showing that participants’ altruistic behavior is more sensitive to moral frames in the context of an Ultimatum game—in which participants have to fear their partner’s rejection—than in the Dictator game in which no punishment has to be feared. Dreber et al.^32^ speculated that “communication, either in form of rejections or in the form of messages, serve to accentuate social norms in the dictator’s mind (perhaps by […] trigger[ing] instinctive fears of retribution), and with more accentuated norms, the labels should start to matter more” (p. 366). In a similar way, the experience-based expectation of being punished may have accentuated the influence of moral labels on cooperation in the present study. Social norms and moral punishment thus may have a mutual influence on each other. The saliency of moral norms may increase the prevalence of moral punishment. Conversely, moral punishment may accentuate the importance of social norms for cooperation and, thereby, the influence of moral labels on cooperation.

Now that effects of moral framing on cooperation and punishment have been established in the present study, it seems desirable to test the robustness and generalizability of these findings in future research. It lies in the nature of empirical research that a single study can only provide tentative answers. While the present study strengthens the hypothesis that cooperation and punishment are based on social norms, independent replications are needed before definitive conclusions can be drawn. Furthermore, the interpretation of the findings of a single study is necessarily limited by the specific choices in the experimental design. First, given that the focus of the present study was to examine how a moral framing affects cooperation and punishment when the participants interacted with each partner only once, the conclusions are necessarily limited to *one-shot* Prisoner’s Dilemma games. In future studies, *iterated* Prisoner’s Dilemma games—in which participants interact with the same partner repeatedly—may allow to track how framing affects cooperation and punishment across repeated interactions. Assuming that repeated games allow participants to apply punishment in more strategic ways to shape the partner’s future behaviors, it remains to be seen whether moral frames have the same or different effects in iterated Prisoner’s Dilemma games compared to one-shot Prisoner’s Dilemma games. Another issue that has to be mentioned here is that the participants interacted with preprogrammed partners which allowed us to gain experimental control over the partners’ choices in the Prisoner’s Dilemma game. This is typical for studies in Experimental Psychology, in which the main purpose is to identify the factors that determine individual behavior so that the partners’ choices are seen as extraneous influences on the participant’s behavior that have to be factored into the design. This approach can be contrasted with Experimental Economics in which the focus of interest often is on dyads or groups rather than individuals. Given that the present results suggest that the effects of moral framing on moral and hypocritical punishment are not affected by whether or not the participants experience punishment by the partners, our intuition is that the same results should be obtained in interactions that involve only human participants (which implies that the behavior of the partner is not controlled by the experimenter), but this remains to be tested in future studies. We also chose to present facial photographs of the interaction partners to make the game more accessible to the participants. This contrasts with research in Experimental Economics in which social-dilemma games are often presented in a more abstract way to avoid distracting participants from the underlying payoff structure. It thus seems possible that the presence of facial photographs may have influenced the participants’ tendencies to cooperate or to punish the partners, which could be tested in future studies. Finally, future studies may explore to what degree the effect of moral framing may differ among different populations. In previous studies, it has been shown that both cooperation and punishment are affected by the participant’s gender as well as by facial characteristics of the partners^37,38^. For example, women were more likely to punish defection than men. Given that these previous studies have employed the labels “I cooperate” and “I do not cooperate” to refer to the behavioral options in the Prisoner’s Dilemma game, it remains to be explored to what degree these effects are influenced by the presence or absence of a moral framing. In general, more research on the robustness and generalizability of the effects seems desirable to arrive at general conclusions about the determinants of cooperation and punishment.

## Summary and conclusions

When cooperation and punishment are studied with social dilemma games in the laboratory, it cannot be presumed that the participants interpret the behavioral choices that are available in these games in the exact same way as the researchers^7^. Labels offer a simple way to communicate a certain interpretation. However, these labels also have a direct effect on the participants’ behavior because the labels change the interpretation of the game. In the present experiments, moral and hypocritical punishment were more likely in a moral-framing condition than in a neutral-framing condition. Furthermore, cooperation was enhanced by moral framing when participants had to fear the partners’ moral punishment in Experiment 2. The results thus strengthen the hypothesis that moral and hypocritical punishment are not only motivated by the experiences of monetary losses but are also influenced by the moral evaluation of the partner’s defection. If these results can be replicated in more ecologically valid settings, this may imply that people can be motivated to cooperate and to punish defection by emphasizing the moral nature of the social dilemmas, for example, by using morally loaded labels for cooperation and defection.

## Data Availability

The original instructions in German with an English translation, a list of the stimulus material (photographs used as partners in the Prisoner’s Dilemma game) and the data analyzed in the current study are available at https://osf.io/kucya/
